# Cannabis Use and Abuse in Nepal: A Review of Studies

**DOI:** 10.31729/jnma.6931

**Published:** 2021-09-30

**Authors:** Dhana Ratna Shakya, Sandesh Raj Upadhaya, Milan Thapa

**Affiliations:** 1 Department of Psychiatry, BP Koirala Institute of Health Sciences, Dharan, Nepal; 2 Health Foundation Nepal, Kathmandu, Nepal

**Keywords:** *cannabis*, *cannabis abuse*, *cannabis use*, *Nepal*

## Abstract

Nepal is a geographically, ecologically, bio-culturally and ethnically diverse country. Cannabis has been a flora, used in various forms and ways and its effect has been variably a matter of concern in Nepal. Perception to its use has also been changing with time with the dynamics of various factors and trends around and in the world. The views of its proponents at times appear with relatively more powerful influence among people though the views of the opponents seem less, and relatively less influential. Proponents advocate for its legalization with highlights of its different uses and benefits whereas opponents, mainly mental health professionals point out sporadically its adverse consequences, mainly in psychological health. The purpose of this review is to explore the existing literature regarding cannabis use and abuse in Nepal. Search for articles for this review was performed in PubMed, Google Scholar, and Nepal Journal Online. We summarize and discuss about cannabis in various aspects in the Nepalese context. The overarching objective is to reflect upon the ongoing debate regarding its harm and benefits, thereby upon the issue of its legalization in Nepal.

## INTRODUCTION

Cannabis is the most widely abused illicit drug globally.^1^ Its source is the plants: Cannabis Sativa and Cannabis Indica^2^ major ingredients being Δ9-TetraHydroCannabinol and Cannabidiol. One study reported Nepalese Hashish containing 17% A9-THC and 9% cannabidiol.^3^

Ethno-medicine is practiced in Nepal for generations^4,5^ and Cannabis has been used for treating diarrhea, constipation and snake-bites in remote communities.^6^ Cannabis was widely used as cattle-fodder and fiber (hemp) in hills, and a few people used it as psychotropic-agents. Its use and attitude have, however, transformed across generations.^7^ Its serious effects were mentioned as madness, weight-loss and decreased semen.^7^ There are case-reports of 'toxic states', cannabis-induced psychosis, schizophrenia precipitation and association with depression.^8^ Despite government ban-1973, it is still being used. In the pretext of ongoing worldwide discussion about decriminalization and legalization, this narrative review tries to give an overview of Nepal's available published evidence regarding its use and abuse.

## LITERATURE SEARCH

This is a narrative review evaluating literature obtained using electronic search in databases of PubMed, Google Scholar, NepJOL published till June 2021. The following keywords were used in different combinations for the search: 'cannabis', 'medicinal', 'cannabidiol', 'THC', 'benefits', 'use', 'indication', 'abuse' and 'harm'. The search strategy used in PubMed was (cannabis[Title/Abstract]) AND (Nepal[Title/Abstract] OR use[Title/Abstract] OR abuse[Title/Abstract] OR indication[Title/Abstract] OR harm[Title/Abstract] OR benefits[Title/Abstract]. A total of 55 articles (case reports, cross-sectional, cohort, and reviews) on cannabis use/abuse in Nepal were included and others as deemed related to cannabis use/abuse in Nepal were cited. The included articles were evaluated by the reviewers to extract relevant information regarding the subject matter.

## GLOBAL CONTEXT OF CANNABIS AB/USE

The least potent form among different potencies in the Indian subcontinent is marijuana and Bhang. Marijuana is consumed mainly as smoking cigarettes (joints) and edible form as bhang during the Holi festival. Intermediate potency (thrice as bhang/marihuana) is ganja obtained from leaves and flowering tops of the male and female plants usually inhaled by pipes. Highly potent (8 times as bhang/marihuana) form is Charas/Hashish obtained from the pure resin of the flowering tops and leaves of the female plant also smoked in pipes.^9^

Over 192 million people, approximately 3.9% of the world population (15-64 years) used cannabis in 2016. Compared to 2006, there is a 16% increase in prevalence. West and Central Africa come first with 34.3 million users (13.2%), followed by North America with 41.5 million (12.9%), Oceania 2.9 million (11%), and least in East and South-East Asia with 9.7 million users (0.6%).^10^ United Nations Drug Report states that the greatest rise in cannabis users from 2010 to 2016, comes from Africa and Asia.^11^ According to the Global burden of disease (2019), cannabis use disorders resulted in 690,000 global years lost due to disability (YLD) accounting for 0.08% (0.05-0.1%) of total global YLDs.^12^ National Epidemiologic Survey on Alcohol and Related Conditions (NESARC) study shows that the probable cumulative chance that cannabis users will transit into dependence in their lifetime is 8.9% and 50% of them will turn into dependence by 5 years.^13^ The evidence shows more cases of psychotic disorder with increasing prevalence of daily use and use of high-potency cannabis in general population.^14^

## CANNABIS AS NEPALESE FLORA

Nepal is known for rich mega-biodiversity possessing a variety of plants with medicinal values.^15^ Since prehistoric times, different plant source medicines have been used in Homeopathy, Ayurvedic, Allopathy and traditional medicine. Bioactive phytochemical constituents (e.g. alkaloids, tannins, flavonoids and some other phenolic compounds) are major contributors to medicinal value. Phenolic compounds possess different biological functions, e.g. antiinflammatory, anti-carcinogenic, anti-aging, cardioprotective, anti-atherosclerotic, and promotion of endothelial function. Saponin compound has been reported to cause tonic and stimulating effects. Among four selected medicinal plants (Ageratum Conyzoides, Eclipta Prostrata, Cannabis Sativa and Woodfordia Fructicosa); the highest amount of phenols, saponin, and essential oil are found in C. Sativa.^16^

Even in rice fields of Nepal, several medicinal plants occur as weeds (grass and broadleaf) due to favorable geographical conditions. These weeds have economic importance due to medicinal, nutritional, industrial use, and as fodder for animals. Cannabis Sativa is among the broadleaf weeds.^17^ Among 104 weed species belonging to 36 families in the rice field of Kathmandu valley that have medicinal values, the leaf of Cannabis sativa var. indica has been reported to have an antitumor effect. As a part of primary health care, traditional practitioners are still using these weeds in curing diseases and proper utilization of medicinal weeds is pointed out as the best strategy of weed management.^18^ Cannabis, both in wild and cultivated varieties has been grown in Nepal for a long time.^8^ It can be found growing wild mostly in the high hills of central, mid-west and far-western parts of Nepal.^19^ Clarke R suggests in a study that northwest Nepal provides a unique example of a basic cannabis agricultural system that ensures all 3 major products-seeds and resin from female flowers, and fiber from stems.^20^

After the imposition of law for punishment for possessing and using the drug, local traders still practice illegal trading in Nepal. Proponents of legalization advocate it as a strong mechanism to stop the illegal trade of medicinal plants and ultimately to increase revenue generation.^21^

## HISTORY OF CANNABIS USE IN NEPAL

The production and use of cannabis products in India and Nepal were well documented during the 19^th^ century by British colonial officials.^20^ James Fisher wrote in 1975 that cannabis was historically cultivated across all altitudes and terrains of Nepal.^8^ Before 1973, Kathmandu was viewed as a 'hippie paradise'.^9^ Cannabis was widely available across the country, and there were even licensed dealers selling hashish in Kathmandu's famous 'Freak Street' ('Jhochhen' in Newari).^22^ A large number of hippies from the western world traveled to Nepal to enjoy the readily available high-quality cannabis. Farmers were free to produce hashish. Nepalese youth gradually started following the hippies' lifestyle and its recreational use grew rapidly. In July 1973, the government of Nepal revoked all licenses to cultivate, buy and sell cannabis, and in September 1976, the Narcotic Drugs (Control) Act 2033 made cannabis illegal in Nepal.^9^ James Fisher points out 3 factors contributing to this government crackdown^8^ : (i) Nepalese alarm that their youth were being corrupted by cannabis; (ii) United Nation's pressure to join other 'respectable' nations in outlawing cannabis; and (iii) US pressure for narcotics control.

## CANNABIS IN NEPAL

The Oldest Vedic Scripture mentions cannabis as a sacred plant due to its close association with Lord Shiva. Spiritually, it is used by Sadhus for meditation and to show their devotion towards Lord Shiva. In social context, people use cannabis at the time of gatherings, festivals, and mourning time to reduce anxiety and stress. Traditional medicine (Ayurveda) uses cannabis to treat ailments like food poisoning, digestion problems, gout and rheumatism and as a pain-relieving agent. It is used also to stimulate appetite and treat sleep problems.^9^ Though illegal, cannabis continues to grow wild, is also cultivated in western and southern Nepal, remains easily accessible throughout the country and its use is largely tolerated without sanction during festivals such as Shivaratri. People use it for smoking and drinking as bhang (ground leaves and flowers from female plants).^9^ Statistics of the Narcotics Control Bureau show cannabis as a consistently and most-seized substance in Nepal.^23^ In 2018, a total of 4,181 kg of cannabis and 1,546 kg of hashish were seized with a slight rise on all accounts from the previous fiscal year.^23^ Data suggest its cultivation to be widespread in Nepal and illegal trade rampant and unabated.^24^

Recent years are witnessing a movement in Nepal to legalize its commercial production, pointing to its potential to become a lucrative cash crop and create employment opportunities. On March 2, 2020, a private bill named, 'Marijuana Cultivation Regulation and Management in Nepal' was registered by a member of parliament Sher Bahadur Tamang at the parliament secretariat of the House of Representatives demanding the legalization of marijuana cultivation in the country.^25^ The advocates of its legalization in Nepal have been trying to convince people that Nepal could benefit immensely from legal cannabis industry, which is expected to reach $146.4 billion by 2025.^23^ However, researchers and commentators have drawn attention to increasing cannabis use among young people in Nepal, and its health and social impacts.^9^ The topic of its legalization to improve country's economy or restrict it to control its potential adverse effects remains a debate to date in Nepal.

## CANNABIS ABUSE IN COMMUNITY

Illicit substances including opioids and cannabis are also highly prevalent after alcohol and tobacco in Nepal.^26^ The 2012 Ministry of Home Affairs survey estimated that among 91,534 'hard drug' users in Nepal, 90.5% used cannabis, and 74.1% combined cannabis with 'tranquilizer'.^9^ Among 393 intravenous drug users (mean age 27.76±4.86 years), who were all male from Kathmandu valley, the gateway drug for more other habit forming and illicit psychoactive substances was cannabis, along with tobacco products and alcohol. This was followed by the use of injection buprenorphine and a mixture of various psychoactive compounds (opiate, benzodiazepine and antihistamines). Intervention must be implemented at the root level to reduce the burden of multiple substances in newbies.^27^

## CANNABIS ABUSE IN CLINICAL SETTINGS

Among 55 all-male cases attending neuropsychiatric OPD of Western Regional Hospital, cannabis (30.9%) was the second most commonly abused illicit drug after intravenous injection buprenorphine (41.8%). Most (86%) were adolescents or young adults; 4% started their use before age 15. The mode of use was smoking cannabis mixed with tobacco multiple times a day.^28^ A retrospective descriptive study of 100 in-patients of De-addiction Ward of TUTH reported cannabis as the commonest initial illicit drug. Among multiple substance users with alcohol as predominant substance (n=6), six were consuming cannabis; opiates as predominant substance (n=34), 15 were consuming cannabis; and benzodiazepine as the predominant substance was also consuming cannabis. Cannabis use is a highly comorbid condition with other substance use.^29^

Among in-patients with alcohol dependence syndrome (ADS) in BPKIHS, 6/60 (10%) reported using cannabis.^30^ A study conducted in seizure patients visiting a psychiatry out-patient clinic in BPKIHS found 4/100 patients reported to abuse cannabis (4%).^31^ One percent of migraine headache psychiatry out-patients^32^ and 4% (3/75) OCD psychiatry out-patients in BPKIHS reported abusing cannabis.^33^

## CANNABIS RESEARCH/LEGAL ASPECT IN NEPAL

Nepal, once famous as a hippie destination, still serves cannabis lovers around the globe. Voices of legalization pour aloud about the potential benefit of legalizing at least for cannabis research in Nepal considering the fact that this country has been a unique natural laboratory because of its diverse bioactive compounds due to extreme ecological alterations in short physical distance and its potential for benefit from at least efforts to study its harm and benefit. Regulated licensed growing facilities and household plant quotas given in specific parts of the country are pointed out to help reduce poverty. For international researchers seeking to collaborate in the name of North-South or East-West exchanges, Nepal has an advantage in terms of relatively lower overhead cost, softer ethics/regulations, and inviting visa options making Nepal an ideal for research about cannabis.^34^

## CANNABIS USE AMONG HEALTH SCIENCE STUDENTS IN NEPAL

Substance including cannabis problem among medical/health science students appears to be a great concern by a relatively fair number of the studies looking into it.^35-47^ Investigation into cannabis ab/use has been a part of overall mental/health and substance research of most of these studies though there are a few studies intensively looking into cannabis use and problem among these students. Most of these studies have heterogeneous subjects (of various programs, years or schools). Although medical students are supposed to be and were relatively more aware about the negative consequences of psychoactive substances, cannabis use was fairly common among them. Most of them looked into its use which ranges from 1% (among nursing students)^39^ to 40% (among those medical students consuming energy drinks^41^ and 35% among dental students)^46^. The physical dependence and other consequences of cannabis among its users were not largely investigated thoroughly. Only a few studies looked into cannabis use disorder/abuse, which reported 2.25%^47^ and other studies looked into problem use requiring medical attention, which reported 9%.^38^ Most of the users were male^35,37-38,40,42^ and had started using the substance after joining the medical college.^38,45^ The prevalence of substance use was seen to have increased from the first year to the final year of medical college.^44-45^ The major motivation behind its use was curiosity, pleasure, peer influence, and relieving stress.^36^-^38,39,40,44,45^ Most of these were cross-sectional studies. Prospective or retrospective studies are necessary to understand the actual effect of cannabis on the health, study and other important aspects of the students.

## AGE OF ONSET AND GENDER COMPARISON ABOUT CANNABIS

The mean age of onset of psychosis for cannabinoids users was younger; 21.80 years (SD=3.36) in comparison with alcohol users 37.95 years (SD=9.5) in a Nepalese study.^48^ A study in western Nepal showed 50% of drug users started using drugs before the age 20.^49^ In most of the studies in Nepal as in abroad; males outnumber females.^37-48,50^ However, in one study among 78 dental students who used cannabis, females outnumbered male users of which 53 (67.95%) were females.^46^

## CAUSES OF CANNABIS ABUSE

The majority of medical students using cannabis gave the reason as pleasure-seeking and experimentation purpose.^45^ Among intravenous drug users, the reason for starting a gateway drug (cannabis) was for the sake of pleasure. A study done among dental students reported pleasure-seeking and difficulty in handling pressure as the cause for the use of substances.^46^ In a study among Ex-client of opioid substitution therapy (OST) in Kathmandu completing their OST, the average duration of cannabis use was 15 years. Many (17/20) users had consumed marijuana to seek pleasure; followed by peer influence, out of curiosity, and least used for getting high. Marijuana is also a popular drug during OST as almost all reported that OST and marijuana in combination reduce the craving for other drugs and at the same time produces the high effect. However, few individuals reported marijuana as a cause of relapse due to increased craving for other drugs. So, the evidence-based fact is inconclusive about using cannabis among OST clients and warrants further study.^51^

## CANNABIS USE; A GREAT THREAT TO MENTAL HEALTH

### CANNABIS AND DEPRESSION

A cross-sectional study was carried out in 42 patients consecutively admitted in the psychiatric ward of Kathmandu Medical College Teaching Hospital and Punarjeevan Hospital. The Majority of them (73.8%) were found to be suffering from depression among which 22 (52.4%) patients used multiple substances like alcohol and cannabis.^52^

### CANNABIS AND ANXIETY

A study from Western Regional Hospital, Nepal showed panic disorder as the most common induced disorder after using cannabis (9/37 patients) who visited psychiatric OPD.^49^ A case-control study was conducted with 30 total cases taking cannabis for a duration longer than 3 months with the frequency of sticks more than 6/months with 30 control groups from medical students. This research did not show any difference in anxiety levels between case and control, whereas alcohol and cigarette use was higher among cannabis users.^53^

### PSYCHOSIS, SCHIZOPHRENIA, ADHERENCE AND CANNABIS

Among 80 first-episode psychosis patients in TUTH, 38 (47.5%) had a history of either current or lifetime use of at least one substance group. Harmful use was present in 42.1% and dependence in remaining. Cannabis was the most commonly used illicit drug. Harmful use was present in 62.5% of cannabis users. Out of 10 cannabis users presenting with psychosis,^2^ were diagnosed with schizophrenia,^1^ with ATPD, and 7 (41%) with Mental and behavioral disorders due to psychoactive substance use, Psychotic disorder was the highest with cannabis among different substances including opioid and alcohol.^48^ A case report of a 20-year-old man who had a brief episode of psychosis upon cannabis withdrawal due to the circumstance of COVID-19 lockdown following the regular heavy pattern highlights the fact that cannabis-induced psychosis is common in countries like Nepal where there is long term and heavy use of cannabis.^54^ In a study from western Nepal, 37 cannabis-using patients were interviewed for DSM-IV (Diagnostic and statistical manual) psychiatric problems. The cannabis-induced psychotic disorder was found in 7 patients. There was a clear relationship with heavy use in which one patient developed within 24 hours of heavy bhang use. They presented with rapid onset behavioral changes like laughing or crying episodes, eating non-edibles, tearing clothes, biting people, and delusions of being controlled and hypnotized. Improvement was seen within 2-4 weeks of stopping cannabis and with a small dose of antipsychotics.^49^ A cross-sectional descriptive study was conducted to study contributory factors for relapse among 95 schizophrenia patients coming to inpatient psychiatry services at Nepal Medical College Teaching Hospital. Substance dependence was a significant factor in the non-adherent group; 8 out of 11 cannabis dependence cases were from the nonadherent group.^55^

### CANNABIS AND OTHER SUBSTANCES

A descriptive study conducted in the de-addiction unit at the Centre for Mental Health (Chautari Nepal Health Foundation) in Rupandehi district of province-5, Nepal looked into the prevalence of psychoactive substance disorders. Out of 387 psychoactive substance users, 34.1% were in the age group 35 years and above and more than half (57.1%) belonged to Brahmin/Chhetri ethnic group. Many substance abusers (44.4%) had initiated the use between the ages of 16-20 years. Choice of substances in younger subjects was opioid (Brown sugar) (31%) and cannabis (16.27%) whereas older clients preferred alcohol.^50^ From 5 different methadone clinics of Kathmandu valley, 84 clients were followed up for 6 months. Out of them, 63 patients remained in the methadone maintenance treatment (MMT) program. One of the significant factors associated with the dropout from the MMT program was poly-substance use in which cannabis was present along with alcohol, heroin, injectables, other opioids and benzodiazepines.^56^

### PSYCHOSOCIAL EFFECTS

The study was undertaken long back by BP Sharma (1975), the first psychiatrist of Nepal, to look into the effect of cannabis use for a long time regularly among 226 individuals, compared with control matched on age and education. The study found cannabis users had poor work records, interpersonal relationships, lack of interest in sexual activity, and overall loss of initiative and efficiency.^57^ Similar replication was highlighted through a case report of a man with a diagnosis of cannabis dependence, cannabis-induced psychotic disorder (CIPD) (later bipolar mood: mania during intoxication and depression during withdrawal), presented in catatonia, published recently from eastern Nepal.^58^

### CANNABIS AND ENDOCRINE SYSTEM

A narrative review on endocrine effects of cannabis in different organ systems based on animal and human studies showed conflicting and contradictory results; some studies showing the lowering level of almost all hormones except cortisol and some studies showing no change in the level of effect. There is also the development of tolerance with repeated administration. Acute and transient effect is seen but long term effect is not conclusive, e.g. cannabis is considered to cause erectile dysfunction but many studies failed to prove the association between its use and lack of erection in men or lack of arousal in women. So, the definite cohort is warranted to establish its true effect.^59^

### KNOWLEDGE, ATTITUDE AND PRACTICE STUDY ABOUT CANNABIS

'Ganjedi' is local term for cannabis users, which literally means a person who cannot be relied on.^57^ Marijuana use in different festivals like 'MahaShiva Ratri' and 'Holi' is a part of rituals based on its belief to be a boon. These socio-cultural factors contribute towards the use of marijuana which ultimately may turn an individual to be a hard drug user.^60^ Screening of medical students for a lifetime and ever use of cannabis revealed 17/61 and current use 2/61; the highest among illicit drugs (heroin and other substances). Their belief system of addiction of substance (tobacco, alcohol, cannabis, heroin and other substance) assessed with Addiction Belief Scale showed majority of medical students believing predominantly the biological model of illness.^61^ A study among Sadhus revealed that only 14% believed Lord Shiva to promote its use. Less educated and Naga sect Sadhus were more likely to view cannabis as a boon of Lord Shiva and provide it to devotees. Providing evidence-based information about cannabis and clarifying misbelief about Lord Shiva and cannabis to Sadhus have been pointed out as one of the strategies of harm reduction in people using substances.^9^

### TREATMENT CONTEXT

For subtypes of persistent headache or post craniotomy pain, cannabinoid derivatives have been mentioned as a potential candidate research molecule for formulating future Post Craniotomy Pain (PCP) management algorithm though Amitriptyline, Gabapentinoids, Carbamazepine or Sodium Valproate, steroid injection, and Botox injection are already available and commonly used medications.^62^ A recent review from Nepal discusses evidence regarding the benefit and harm of cannabis in various neurological, psychiatric and other diseases with the assertion that the adverse effects are adequately evidence-based and are at times serious, especially for psychological health.^2^

In 2010, World Health Organization (WHO) introduced Mental Health Gap Action Programme (mhGAP) for mental, neurological and substance use disorders (MNS) in a non-specialized health setting. The main aim is to enable non-specialists in primary healthcare to detect and treat priority MNS disorders and make appropriate referrals to a higher system of care. Under mhGAP-Intervention Guide training module locally adopted also in Nepal, Mental and Behavioral Disorders due to Substance Use, Cannabis has been discussed with different types of cannabis available. Different subheadings starting from introduction to different substance use, assessment, management, and follow-up along with emergency presentation are discussed.^63^

## CANNABIS; A HOT DEBATE ALSO IN NEPAL

Cannabis use in Nepal is intertwined with religious, cultural, economic since ancient times and later political perspectives. Amendment of the Narcotics Drug Control Act 1976 in Nepal prohibits the sale, cultivation and use of cannabis. UN 'Single Convention on Narcotics Drugs 1961' was also signed by Nepal in 2003. Nepal has also been swept by the wave of hot debate ongoing in the world about decriminalization and legalization of cannabis. With a vote of 27 in favor, Nepal also voted in December 2020 to remove cannabis from schedule-IV of the 1961 convention opening the avenue for its therapeutic use. A private bill was also registered at the House of Representatives in Nepal for the legalization of marijuana, mainly for medical use and export. Voices are outpouring in media to decriminalize cannabis use as the democratic generation doesnot want interference from the government with an individual's choice of what goes into their body as long as it does not harm others.^34^ They view cannabis cultivation would boost the country's economy.^64^

## WAY FORWARD

Regarding medicinal use, only a few studies show some benefits in certain conditions (pain, nausea, vomiting, spasticity); mostly inconclusive. On the other hand, the current understanding of cannabis use in any form (medicinal or recreational) showed more evidence for harms than therapeutic benefits in the majority of health conditions mainly from the perspective of mental health effects and serious conditions (e.g. cardiac dysrhythmia).^2^ Serious considerations and more study are warranted in this field before any step is taken and information about its use to be provided by policymakers to care providers, people and other stakeholders as public/individual health are of paramount importance.
